# “Navigation to prioritizing the patient” – first-line nurse managers’ experiences of participating in a quality improvement collaborative

**DOI:** 10.1186/s12913-020-4918-z

**Published:** 2020-01-22

**Authors:** Berit Mosseng Sjølie, Trude Anita Hartviksen, Terese Bondas

**Affiliations:** 1 Nord University, Faculty of Nursing and Health Sciences, Storgata 105, N-8370 Leknes, Norway; 2 University of Stavanger, Faculty of Health Sciences, P.O. Box 8600, Forus, N-4036 Stavanger, Norway

**Keywords:** Quality improvement, Quality improvement collaborative, Network, First-line nurse manager, Nursing leadership, Patient outcomes, Bondas’ theory of nursing leadership, Content analysis

## Abstract

**Background:**

First-line nurse managers are central to quality improvement work when changing work practices into better patient outcomes. Quality improvement collaboratives have been adopted widely to support quality management in healthcare services and shared learning. We have little knowledge of the first-line nurse managers’ own perspectives concerning their need for support and knowledge in quality improvement work. Therefore, the aim of this study was to gain understanding of first line nurse managers’ experiences in leading quality improvement work in their own organization when participating in a quality improvement collaborative.

**Methods:**

An interpretive approach was chosen following Graneheim and Lundman’s qualitative content analysis. Data was collected through three focus group interviews with first-line nurse managers representing different workplaces: the local hospital, a nursing home, and a homecare service in a rural area of Norway.

**Results:**

“Navigation to prioritizing the patient” emerged as an overarching metaphor to describe the first-line nurse managers experiences of leading quality improvement work, based on three themes: 1) fellowship for critical thinking and prioritizing the patient; 2) mastering the processes in quality improvement work; and 3) the everyday reality of leadership as a complex context.

**Conclusions:**

A quality improvement collaborative encompassing knowledge transfer and reflection may create an important fellowship for health care leaders, encouraging and enabling quality improvement work in their own organization. It is crucial to invite all leaders from an organization to be able to share the experience and continue their collaboration with their staff in the organization. Continuity over time, following up elements of the quality improvement work at joint meetings, involvement by users, and self-development of and voluntary involvement in the quality improvement collaborative seem to be important for knowledge development in quality improvement. The supportive elements of the quality improvement collaborative fellowship were crucial to critical thinking and to the first-line nurse managers’ own development and security in mastering the quality improvement work processes. They preferred prioritizing the patients in quality improvement work, despite haste and obstructive situations in an everyday context.

## Background

Scientific evidence of medical treatment and nursing care have increased during the last few decades [1], including changed circumstances for patient involvement [2] and interdisciplinary collaboration [3]. This makes it reasonable to expect both new challenges and opportunities when planning, developing, and ensuring nursing care [4]. First-line nurse managers (FLNM) work closely with everyday clinical practice, and play a crucial role in leading and coordinating quality improvement (QI) work [4]. We use the term “quality improvement work” (QI work) and understand this as a *change to work practices or work organization that results in better patient outcomes* [5]. To accomplish this, a greater understanding of nursing leadership and management in improvement work is needed [6]. The involvement of FLNMs is central to the development of structures and cultures to improve patient safety and achieve sustained quality in nursing care [1]. There is growing awareness of the complexity perspective in healthcare among scholars, academics, practitioners, and policy-makers [7] and of how different types of leadership influence outcomes [8]. Within healthcare generally and QI work specifically, adaptive leadership that regards human interaction [9] and is responsive, flexible, and open to change [4], seems to be more useful than leadership based on control [4].

Quality improvement collaborative (QIC), described as organized, multifaceted quality approaches to a given healthcare topic of limited duration [10], have been adopted widely to support quality management in healthcare services and shared learning about QI work [11]. We have little knowledge of the FLNMs’ own perspectives on their need for support and knowledge in QI work [1]. The focus of this paper is therefore FLNMs’ experiences of leading QI work in their own organization when participating in a QIC.

### Nursing leadership development

It is suggested that a comprehensive network of processes is needed to support the continuing development in nursing leadership, and is best suited to take place within the organization [12]. Studies show that FLNMs who play an essential role in quality management need contextual support to develop the capacity and capability for leadership [13]. Promoting a supportive working atmosphere can contribute to professional networking and collaboration, where FLNMs feel free to share their vision and purpose, thereby developing decision-making, managerial, and leadership abilities [14]. It is likely that collegial collaboration as a supportive element helps FLNMs improve their ability to cope at work and enhance decision-making skills [8]. Task support, referred to as the sharing of assignments and exchange of ideas among colleagues, was important to complete work, make decisions, and solve problems in a group of nursing home leaders [15]. The QI work supporting development teams required integration of both a caring leadership culture and caring actions through humane and humble leadership [16]. Supervision seems to help FLNMs prioritize and set boundaries, and increases their clarity, certainty, and consistency in leadership activities [3]. If FLNMs are well-supervised in their decision-making, they may be empowered to take control of their work and initiate QI work, while also utilizing the strengths of other employees [14]. However, FLNMs do not want employees to regard them as controllers [17]. A caring culture of nursing leadership facilitates the best patient care by trusting the professional staff and their knowledge [18]. The reflective and supportive characteristics of supervision can promote FLNMs’ individual development, but also the development of leadership, resulting in improved patient care [19]. Learning activities based on transformative learning may promote FLNMs’ individual leadership development [20].

### Quality improvement collaborative

QIC brings together groups of practitioners from different healthcare organizations to work in a structured way to improve one aspect of the quality of their service [10]. Several studies on QIC report that evidence of improvement in patient outcomes is encouraging, but must be interpreted cautiously since the reports do not meet established quality and reporting criteria [11]. Successful improvement interventions seem to be related to the strength of the supporting evidence base of the topic, the simplicity and practicality of the intervention components, and contextual factors [11]. Generally, relationships that foster institutional work, and aligning QI work with professional logic, together with work structures that empower employees, may influence how employees engage in QI work [21]. It is suggested that more often the reason nurses and doctors get involved in QI work, it is because the work is aligned with what they perceive to be important [22]. Valid descriptions of the conditions influencing QI work may be important to understanding the factors affecting the success of QI work [5], including QIC as a factor.

The FLNMs are central to QI work when changing work practices into better patient outcomes [4]. The numbers of interacting components in QI work in the nursing field require flexibility in design and implementation [4]. It is suggested that a comprehensive network of processes is needed to support the development of this type of work, and that sustained development needs to be workplace-related [12]. QIC is frequently used to support QI work despite limited research [11]. More understanding is needed about the meaning of QIC participation in the leading of QI work.

### Purpose

The purpose of this study was to gain deeper understanding of FLNMs’ experiences of leading QI work in their own organization when participating in a QIC. Two research questions were posed: 1) How did FLNMs’ experience of participating in a QIC contribute to leading QI work in their own unit? 2) Did FLNMs’ experience of contextual factors contribute to or become an impediment when leading QI work?

## Method

### Design

We found a qualitative and explorative approach most suitable, allowing a determination of FLNMs’ own experiences and views without predefined categories. Data was gathered through three focus group interviews, and analysis was performed using the qualitative content analysis with an interpretative approach [23]. In this study, the phenomenon was described in a conceptual form and the data was viewed as representations not of physical events, but as texts and expressions created to be heard, read, interpreted, and acted on for their meanings [23].

### Study setting

The setting for this study was a QIC in a rural part of northern Norway. Contrary to the classical approach of a QIC within a given healthcare topic of limited duration [10], this QIC focused on knowledge development within the general topic of improvement strategies for an undecided duration.

The QIC consisted of 54 participants who met three to four times each year to: 1) share the development of leadership and improvement knowledge; 2) receive guidance for the practical performance of improvement practices; and 3) social networking. The meetings consisted of short lectures and group workshops within and across organizational borders in different conference locations in the participating municipalities. The researchers’ access to the QIC was facilitated, since both the first and second researcher participated in the QIC from the start. This QIC was initially financed by the County Centre for Development of Home Care Services, the participants’ organizations, and the County Council. The QIC included 40 first- and second-line leaders from rural municipalities, ten first- and second-line leaders from a local hospital, three lecturers from a local university department, the manager of the County Centre for Development of Home Care Services, and one participant representing the recipients of public healthcare. The Norwegian Knowledge Centre for Health Services served as the coordinator of the meetings’ schedules and the thematic structure of the daily programs. All participants had a clinical background, mainly as nurses, but there was also one social worker, three physicians, and one occupational therapist.

The theoretical perspective of the QIC was inspired by Illeris’ [24] perspective of transformative learning. Illeris [24] combines a variety of learning theories into a comprehensive framework, specifically aligned to adult learning. This framework explains all learning as both individual and social. The individual receives impulses through social interaction, incorporated as internal interpretation and acquisition. It is suggested that transformative learning involves changes in the learners’ perspectives as a result of critical reflection, open discourse, and the implementation of new understanding in practice [25].

### Participants

To capture various perspectives on the FLNMs’ experiences of participating in the QIC [26], invitations to join the focus group interviews were sent by email to FLNMs representing three different workplaces; the local hospital, one of the nursing homes, and one of the homecare services. The selection criterion was that the workplaces had participated in the QIC for more than 1 year, and that the workplace currently had at least 5 FLNMs participating in the QIC. These criterion considered, 26 of the total 54 participants in the QIC were eligible to be invited to the focus group interviews.

Of the 26 invited, sixteen FLNMs participated, which is a 62% participation rate. The FLNMs were divided into three focus groups representing their workplace, thus, each workplace was represented by five to six FLNMs. Still, the total number of participants in the focus group interviews was seventeen, due to one user representative who took part in all focus groups. Thus, each focus group consisted of six to seven participants, all groups including the same user representative (Table 1). The participants were between 29 and 68 years of age and 76% were women. All groups had one male FLNM participant, as well as the same male user representative, totaling two male participants in each focus group. Thus, of the seventeen participants, four were male. Work experience as a FLNM ranged from 1 to 28 years. Table 2 shows participants’ characteristics.

**Table 1:** Participants divided into the focus groups

Participants	Focus group 1 FLNMs from the local hospital (H)	Focus group 2 FLNMs from the municipal long-term care services (LTS)	Focus group 3 FLNMs from the municipal homecare services (HS)	Total
First-line nurse managers	6	5	5	16
User representative	1	(1)	(1)	1
Total	7	6	6	17

**Table 2:** Participants’ characteristics

Gender	
Male	4 (24%)
Female	13 (76%)
Age	
Median	52 years
Mean	48,4 years
Spread	29–68 years
Total work experience as FLNM	
Median	7 years
Mean	9,8 years
Spread	1–28 years

### Data gathering

The interview guide (Additional file 1) for the focus group interviews was developed by the first and second researchers, based on existing literature and input from FLNMs and user representatives involved in the QIC. The questions in the interview guide were framed to stimulate dialogue and reasoning from a critical and reflective perspective [27]. The data were gathered in December 2014, through three semi-structured focus group interviews in quiet locations, for approximately one and a half hours [26]. Together with two of the researchers, the respondents were offered lunch free of charge before the interview started, as a token of goodwill and for small talk before the interviews. Two researchers, both female, who alternated positions as moderator and assistant moderator, conducted the interviews. The moderator asked questions and the assistant moderator had the responsibility of audio recording the focus groups and taking notes that included body language and other visual cues, including group dynamics [26]. The first author conducted one of the interviews, while the second author conducted two interviews. The interviews were audio recorded and transcribed into verbatim text. The transcribed materials included 87 pages of text, containing 31,987 words.

An interview constitutes a specific setting for the dialogical production of personal narrative and social life [26]. Focus group interviews are appropriate for exploring a new research field and the strength of relying on the researcher’s focus is the ability to produce concentrated amounts of data precisely concerning the topic of interest [28]. A strength for focus group interviews is their reliance on interaction in a group that reveals spontaneous expressive and emotional viewpoints. However, the group may influence the nature of data by withholding private meanings or expressing more extreme views than they would in private [28]. Similarly, the presence of the researcher as a moderator in a focused discussion of a preselected topic may lean the data toward the stated poles of the continuum [28]. The researchers tried to create an open and inviting dialogue.

The focus questions of the interviews included: How would you describe the benefits of participating in this QIC? Is there a difference between how you practiced leadership before and after joining the QIC? What influence does participation in the QIC have on patients and employees? Probing questions were asked to increase the depth of the interviews. All participants, including the user representative, contributed as much detailed information as they wanted, and at the same premises. The participants followed up on each other’s statements in a fluent conversation. The interviewer added complementary questions, such as: Can you add some examples? How did this happen? How did you know this? What was less or not useful? How could this be changed?

### Ethical considerations

This study is part of the Centre for Development of Institutional and Home Care Services Nordland project (13211141), and was supported by the Nordland County Council (project 18-15-0041). The study was submitted to the informal notification test provided by the Norwegian Centre for Research Data [29] and was not found to be subject to notification. The study did not contain patient information or any directly or indirectly identifying information about the participants.

The following ethical guidelines were used: 1) Participants were informed verbally and in writing about the purpose of the research and their right to make independent decisions without negative consequences, including withdrawing at any stage of the research. 2) Participants were not pushed to give information. 3) Participants gave written informed consent to participating. 4) Ethical challenges related to conducting focus group interviews when the researchers and participants knew each other were considered and found not to be problematic by the participants, as the group expressed a high degree of trust during the interviews [30].

### Data analysis

The analysis followed Graneheim and Lundman’s [23] qualitative content analysis. The process involved a back and forth movement between the whole and parts of the text, and between preunderstanding and understanding [26]. After transcription, the text was read through several times to obtain a sense of the whole. The text was then sorted into three content areas, understood as specific, explicit areas of content identified with little interpretation [23]. The first and second author did the reading and sorting individually, but worked together to decide the content areas. The third author read the interviews and discussed and validated the findings. This reflection and discussion resulted in agreement on three content areas that gave a basis for sorting the units of meaning. Subsequently, the text was divided into units of meaning, which were condensed. The condensed units of meaning were abstracted and labelled with codes. The codes were then sorted into categories and sub-categories, based on comparisons regarding their similarities and differences. Lastly, themes to express the latent content of the text were formulated [23]. Opportunities for participant checking of findings were provided throughout the analysis process. See Table 3 for illustration of the analysis process, from the units of meaning to the categories and themes.

**Table 3:** Illustration of the analysis process, from the units of meaning to the categories and themes

Theme 2: Mastering the processes in improvement work			
Category	Sub-category	Code	Quotations
Becoming proficient	Transformation of knowlegde	Knowledge development	*Compared to a regular study you go into for a certain time, and when you’re done. It’s assumed that you use this knowledge* (LTS4) *From the first convention to the last, the last one, I might have, I mean here it is, I’ve grown, something has happened here* (HS4) *Become more observant of how important it is to have knowledge-based, for example, procedures* (H4)
		Future-oriented	*And then I think ‘wow, I’ve been through that, I’ve learned that, I’ve seen that, I’ve heard about that’, and then you use it* (H6) *And so, many times, I think that it might as well just be there, because it will probably come up some time* (HS3)
		Recognizable knowledge	*Because we do have worked for a long time in this way, before we started in the network* (HS5) *Really, I don’t think that we have done it differently* (LTS2) *We can sit in meetings and see [Name] present this as news, and we who are here, of course, find this a little funny* (H6)
		Importance of reflection	*I’m thinking about the consciousness-raising, that it’s important to put words on things, and let them (the employees) discuss the pros and cons, or ask questions. I realize something has happened. They answer themselves.* (LTS1)
	Understandable processes	Processes related to the perspective	*When we understood Evidence Based Practice, then I thought: Now I gather the details together into a whole* (H4) *We get the latest in a way, and that’s thrilling* (LTS5) *There are elements here that makes you think a certain way, that you can bring with you, which covers a lot of what you would need to keep in mind when working with improvement work as a leader* (HS2)
		Processes related to the system	*We’ve worked with processes before, but back then we didn’t have the same understanding of what was necessary. To go through this process* (HS5) *What I’ve learned? That’s this takes one’s time* (H1) *There’s a lot of things to keep in mind, it’s very wide, you have to think about every little thing that happens in the department, to what person you hire* (H3)

## Results

**Fig. 1:**
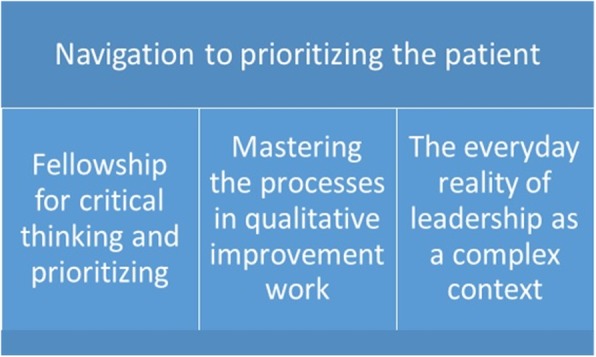
First line nurse managers’ experiences of leading quality improvement work when participating in a Quality Improvement Collaborative

The FLNMs’ experiences of leading QI work in their own organization when participating in a QIC is described by an overarching metaphor and three themes. The overarching metaphor “*Navigation to prioritizing the patient*” is based on the following three themes: 1) Fellowship for critical thinking and prioritizing the patient; 2) Mastering the processes in improvement work; and 3) The everyday reality of leadership as a complex context for QI work. (See Fig. 1.) In the citations, “H” is used for FLNMs from the local hospital, “LTS” for FLNMs from municipal long-term care services, and “HS” is used for FLNMs from the municipal homecare.

### Fellowship for critical thinking and prioritizing the patient

The participants described their experiences of how participating in a QIC changed their way of thinking. A new, critical way of thinking evolved that put the patient in the foreground of the QI work. The participants explained that the QIC made them more aware of the importance of patient involvement and underlined that this involvement contributed to the success of nursing. Patients’ feelings of safety, satisfaction, and involvement, and adapting nursing care for the patients and their relatives, were issues when the participants talked about quality of care. Fellow participants in the QIC seemed to have had a supportive impact on the participants regarding improved patient involvement and QI work in general. This support gave the participants a feeling of confidence, contributing to a shared understanding of nursing leadership and quality management. This theme encompassed two categories: 1) an awareness of responsibility for patient safety; and 2) a supportive platform for development and security.

#### An awareness of responsibility for patient safety

The participants described how, as leaders, they played a vigorous part in improving patient involvement. The data revealed that the participants were in no doubt about their responsibility for the quality of care in their own department. It seemed this awareness increased after joining the QIC:

*“I feel therefore it is more responsibility, much more responsibility than … ., I’m more conscious about it now than before, that it is my responsibility to bring forward what is the best for the patient. That I think a lot about.”* (LTS4)

One of the participants expressed this responsibility, and not being sure whether their service was up to standard:

*“I feel more pressured, or I don’t feel certain that the care we provide is good enough, that it is evidence-based good enough, meaning that the patient gets the new and updated, and that the treatment is correct.”* (H4)

The participants described in different ways how patients should not only be safe, but also feel safe. Participation in the QIC clarified that a sense of safety was essential for the patients. They considered patient information to be vital, and how this information was presented was essential for the patient’s sense of safety:

“*That he [the patient] feels that we know what we are doing and that it is thought through.”* (H6)

When it came to communication with the patient, one of the participants told us about how the department had changed:

“*Now we have assigned resource persons, we have informed and comforted them [patients] and … . Perhaps we would have gone straight ahead, if we did not have this [QIC]”.* (H6)

The leaders from the local hospital emphasized an increased awareness of the national campaigns as being vital for patient safety:

*“I guess this is the new way we handled these campaigns. That if we had not joined this [QIC] we would have handled the patient safety campaigns differently.”* (H2)

Taking part in the QIC increased the leaders’ awareness. It was not enough that the staff assured the procedures so that the patients were handled safely. The patients also had to be assured that the treatment was correct, according to the updated knowledge.

The participants clarified that patient involvement in QI work is essential to improving quality. They reminded us that:

“*Only the wearer knows where the shoe pinches.*” (HS5).

They considered it essential to reveal this knowledge in the day-to-day work. One of the participants underlined this when describing the use of questionnaires for a patient survey:

*“The only way to get feedback is to ask patients continuously: ‘How do you feel about the service we are providing’?”* (H6)

Participating in a QIC had given the participants several ways to involve patients and listen to their relatives.

#### A supportive platform for development and security

The QIC was experienced to give the participants valuable support, as, for example, confidence in their own leadership and in their personal development in the leader role when planning QI work. One of the respondents explained that the QIC was her only meeting point related to this aspect of leadership, since other meetings focused on reporting and economic management. She described it as the only leadership support she had, and that it was so valuable she was willing to pay for it herself.

*“I’ll have nothing if I lose this. If this is as much as I’ll get during a year, well, then I’ll pay for it myself. But that gives them such a bad conscience, that they pay for it anyway. And I mean that seriously. Because this is the only thing I have, I have nothing else.”* (H6)

Another pointed out there might not have been any awareness of the necessity of QI work if they had not participated in this QIC:

*“If I had not been a part of this network, then I would have neither improvement work in mind, nor the idea about evidence-based practice.* (H4)

Participation in the QIC gave the FLNMs confidence to perform QI work:

*“Concerning the focus on the daily work, both small and large projects have been … . Surely, we still have acquired some changes, but I think we would not have been where we are today without … [QIC]. Neither me as a leader, nor the entire group of leaders. Both the theory about microsystems and … , or me as a person.”* (HS2)

*“When we have been at the network meeting and come back, it is fresh in the head, and it is easier to work together with the colleagues who have been there.”* (LTS2) *“I agree*,” one of the others in the same leader group added: *“Exactly, it is important that we all have come this far, somehow we have been through the same.”* (LTS4)

The participants considered the shared understanding they achieved through the meetings to be valuable, since it was in accordance with their day-to-day challenges. This was of practical benefit in the day-to-day work and, over time, gave them the ability to see organizational challenges in a different light.

*“I think it’s more important that there is a kind of network that does something with both the theoretical part in leadership, but also something that makes me able to think about how I can connect this to where I am, I think, according to the things I’m doing. And I think that’s good”* (LTS1).

One of the participants said she was so convinced of the advantages of the QIC that she had nearly threatened one of her colleagues to join the meetings. She had said to her:

“If you want to join me in going forward you must follow along on what we’re doing when it comes to improvement work, (and) the conceptions around it. … Like I said to her, you put aside a few days of your life in one year, so you’re left with 360 days to operate, and then you can join me for four days as leader and learn about improvement work. And this is a deal she can live with, and that’s good” (H6)*.*

The participants pointed out their own personal development. One of them expressed it this way:

*“And I think this is not just a network that entails improvement work … I mean, you learn about yourself, and stuff like that. You become aware of aspects of yourself that you can change … and I think that’s good, too”* (LTS4).

The participants had also become more aware of how their own personal capacity as a leader gave them a sense of security in their leadership role. They told us they were working determinedly on discovering their own personality, and now saw their own personal advantages and disadvantages in a better way. Moreover, the participants summarized that the QIC had given them a sense of confidence in their own decisions when they made changes in their own departments. One of them put it this way:

*“I feel that by having gone along with this network, I mean, in the past, as a leader, if you wanted to make changes, you might have been less secure without being aware of it.”* (HS4)

### Mastering the processes in QI work

The methods and techniques for QI work that the leaders learned in the QIC gave them knowledge that could be applied to the QI work, and acknowledgment of how they directed the QI work in their own department. Overall, it gave them a sense of becoming proficient in the practical day-to-day QI work in the field of nursing. The participants said they took responsibility for patient safety and improved services. Nevertheless, they reflected that employees needed to become capable of performing QI work. This theme encompassed three categories: 1) developing a common language and expression of what to achieve in quality improvement work – giving strength; 2) becoming proficient; and 3) the importance of support and involvement when intermediating employees’ responsibility for QI work.

#### Developing a common language and expression of what to achieve in QI work – giving strength

Participants gained a mutual understanding of QI work, both among the participants in the QIC and their own local group of leaders. One participant told us she was amazed by the similarity of challenges across the healthcare systems. Another participant talked about her fellow members in the QIC and the mediation:

*“And it’s not like … ´I’ve accomplished this, but I’m not going to share it with anyone´ Because they share - it’s part of the improvement work, you could say. It’s like this: they share what they have accomplished, and they share what they have not accomplished.”* (LTS1)

Another participant put it this way when he talked about being motivated for QI work:

“*You go there and you kind of get fired up, and then you come home, and you’re still fired up and if you had never gone there, then you might not have become fired up.”* (H6)

At the same time, the participants considered it very important that the local group of leaders met together in the QIC.

*“It’s important that we have kind of come just as far, that we, as a leader group have heard and been through the same.”* (HS5)

*“I feel that when we speak about things, among the three of us, I mean, that there is a sense of understanding, an insight, that these kinds of things take time – or that we do this this way, or some other way.”* (H3)

#### Becoming proficient

The QIC helped participants better understand and handle the requirements in the QI work processes. Participants expressed a lack of education regarding their own subject area, i.e., leadership and QI work. Nevertheless, compared to the QIC, they realized that regular education would not have prepared them sufficiently in how to use the knowledge in a practical way.

The QIC may thus have functioned as a better support for developing leadership in QI work. The knowledge the participants gained regarding new methods and techniques was experienced as future-oriented. They emphasized that they were pleasantly surprised by the effective output when they faced a challenge in their everyday work.

*“And then I think ‘wow, I’ve been through that, I’ve learned that, I’ve seen that, I’ve heard about that and then you use it.”* (H6)

Despite earlier QI work experience, the QIC participation seemed to have helped the leaders better understand and meet the requirements in the QI work processes.

*“We’ve worked with processes before, but back then we didn’t have the same understanding of what was necessary.”* (HS3)

Participation in the QIC gave them evidence-based knowledge and connected this knowledge to the day-to-day work. This gave them effective output and an overall picture of the QI work.

*“We get the latest, in a way, and that’s thrilling.”* (LTS5)

#### The importance of support and involvement when intermediating employees’ responsibility for QI work

Employees’ sense of confidence and responsibility for QI work depended on guidance and attendance of QI work, as one of the participants explained:

“*The starting point was that the patients were to have as good a health service as was possible through the people employed there.*” (HS3)

One of the participants emphasized that the leader’s role was to give the employees the appropriate tools to perform this work in a safe atmosphere, and described how she put the employees in this position.

*“I think it is my responsibility that I prepare for them [the employees], so that they will give the best nursing care.”* (H4)

She told us she considered herself a supervisor for the definite purpose of assuring the staff.

*“I use the time I get with the staff to aim at or point out the importance of evidenced-based practice. So they feel safe when patients ask questions, and they can answer and be sure that what they say is correct, or that the information they give is up to date.”*(H4)

The participants expressed that the QIC had improved their opportunities to carry out QI work together with the staff.

### The everyday reality of leadership as a complex context for QI work

The participants described the context for QI work as days characterized by interruptions, and in contrast to the wishes expressed, the participants explained that they had to establish appropriate routines for QI work. The interruptions obstructed a comprehensive view of QI work, caused prioritizing dilemmas, and resulted in haste. This unavoidable everyday reality seemed to become a complex context in which it was challenging to undertake QI work. Despite this situation, the participants still drew attention to patients’ needs and involvement. This theme encompassed two categories: 1) despite good intentions, obstructive situations influence the ability to carry out the QI work; and 2) haste overshadows thoughtful actions.

#### Despite good intentions, obstructive situations influence the ability to carry out QI work

Obstructive situations influenced the participants’ ability to realize the good intentions to stay focused on improvement efforts, because other tasks suddenly got in the way, and because dilemmas had to be prioritized since senior management gave the plans for QI work less priority than financial management.

*“The good intentions we have are eaten away, because suddenly some mails tick in that should have been replied to yesterday, or last week, and suddenly you’re sitting up late at night thinking about things that were promised, and ‘oh, this isn’t what I’m supposed to be doing’”.* (H6)

The participants compared their current QI work with their previous, more fragmented work when they had not seen the entire picture of the possibilities and connections of QI work. The participants expressed the development in the QI work as a better way of thinking about solving problems that included the patient in a more central way. One of the participants gave an example of how they systematically examined how to reduce the personnel rotation frequency for patients in homecare services:

*“Speaking of this rotation scheme and keeping as few patients as possible per caregiver. That was the kind of goal we set for ourselves, to have an emphasis on the users.”* (HS3)

Another participant in the same group added:

“*Yes, emphasis on the user all the way, and we’ve had that, we’ve had regular meetings on who should do what – this is thoroughly thought through, but it’s probably as [name] says, hurry up slowly, find the best solutions.”* (HS5)

Nonetheless, one of the them still found it challenging to stay focused on QI work because of its complexity.

#### Haste overshadows thoughtful action

The participants experienced both a lack of time for QI work and a lack of time generally when several problems needed to be solved. This led to hasty solutions that did not necessarily give permanent results. Nonetheless, the participants considered haste to be unavoidable. This occurred when the participants talked about acting too quickly when they encountered a challenge.

“*Well, it is somehow easier said than done. Then it goes too quickly, because you think, oh, I need to do something.”* (H1)

Another participant put it this way:

*“And so, I make a phone call to get it done, and then the next day you realize that this isn't working, so I make another phone call ... Still, it doesn’t work ... How many phone calls should you make?”* (H6)

The participants often used the word *firefighting* to describe being in a hurry, particularly for before they joined the QIC. When the researcher asked what the participants meant by *firefighting,* they answered that some problems needed to be solved immediately.

## Discussion

In this study, *“Navigation to prioritizing the patient”* emerged as an overarching metaphor to describes the FLNMs’ experiences of leading QI work when participating in the QIC. Navigation deals with plotting, ascertaining, and directing the course of a ship. The FLNMs want to ensure their patients receive a safe voyage, where safety and involvement in medical treatment and nursing care are well taken care of by a competent crew. In the QIC, the FLNMs experienced a safe harbor with a port for uploading new supplies, sorting out ropes that spun during the crossing, and gathering together with other sailors to prepare the next sailing route. Findings indicated that a fellowship for critical thinking and prioritizing the patient was created in the QIC. The participating leaders experienced an awareness of responsibility for patient safety and a supportive platform for the leaders’ own development and security. Metaphorically, FLNMs had good knowledge of the crossing, but realized that they regularly had to go back to the port to bunker, for both supplies and new knowledge about the constantly changing circumstances in the sailing route. Still, many obstructions made maneuvering the ship challenging. Our results indicated that mastering the processes in QI work was manifested by becoming proficient, developing a common language and expressions of what to achieve when intermediating to employees their responsibility for QI work. Contextual factors contributed to or became impediments to the QI work in the everyday reality of leadership, as a complex context for QI work where obstructive situations influence the ability to achieve good intentions, and haste overshadows thoughtful actions.

The participants described the QIC as a place to return to, almost physically. It was experienced as a safe place of fellowship, recognition, support, and confidence, like a safe harbor before crossing the ocean. The repetitive dockings seemed to have changed the participants’ way of thinking, to a more critical way that brings forward the patient in QI work. The data confirmed that the participants experienced a change in their priorities towards a more patient-centered focus in QI work, with a caring leadership culture. These results adds to Bondas’ [16] findings concerning the integration of both a caring leadership culture and management actions committed to the best patient care, in her investigation of the development of self-organizing ad hoc teams for innovative nursing care.

The results also add to the understanding of the QIC as a port of call, used for repair, uploading, cleaning, and painting the ship. The participants described how they mastered the QI work processes in the everyday reality of leadership as a complex context for QI work. Even when they were interrupted they drew attention to the patient. They had experienced being in a port, receiving personal and practical support, and then going back to sea to perform QI work in their own department, with the patient as the focus of their attention. This adds to the findings in a study that show the power of the reflective nursing and caring science based clinical supervision to enhance nursing care. It looks as though the supervision in the QIC may be compared to group supervision in a clinical environment [19].

Several studies have found the supportive elements of the supervision of nurses in administrative and leadership positions to be important for reflection upon their decisions [3, 15, 16, 19]. The participants in this study expressed dependence on the QIC to manage the demands of the day-to-day work. This dependence can be explained by the supportive elements of collaboration between colleagues. These results add to Sirola-Karvinen and Hyrkäs’s [3] findings concerning how the restorative function of clinical supervision promotes ways of setting boundaries and understanding leadership. Collegial collaboration seemed to help the participants in this study prioritize and, according to Sirola-Karvinen and Hyrkäs [3] study, increase in clarity, certainty, and consistency in leadership activities. In addition, it was likely that collegial collaboration as a supportive element helped the FLNMs in this study improve their ability to complete QI work, make decisions, and solve problems in their own department. Successful decision-making processes ultimately improve service delivery and patient outcomes, depending on the existence of a supportive environment [15].

The analysis suggests that joining the QIC may affect the FLNMs’ development in QI work in a way that ensures the quality of care in their department. In contrast to the past, when they did not participate in the QIC, they now take personal responsibility to bring forward new knowledge about nursing care in the best interests of the patient. Many other studies confirm that nurse leaders feel concern - and consider themselves responsible - for the development of nursing care in their own unit [1, 17]. The development in this study was that awareness of personal responsibility seemed to be strengthened by knowledge of the importance of the patient’s involvement in the development processes. The patient orientation within QI work has increased in recent years, and there is discussion of whether the change is due to patient pressure or decisions made by policymakers [2]. This study indicates this might be related to a caring approach in QI work, likewise shown by Bondas [16]. Despite the everyday reality of interruptions and haste, the FLNMs in this study drew attention to the patient. Studies show that when nurses are involved in QI work, this is most often because the work is aligned with what they perceive to be important [22]. According to Bondas [17], the core performance of the nurse leader is to connect the main functions in the unit with the care of patients by directing nursing care and focusing on care outcomes.

The FLNMs in this study expressed how they gained inspiration for QI work, including the knowledge to fulfil the QI work processes’ requirements. These statements may be related to the self-development of this QIC and the voluntary involvement, and the teaching in the general topics of improvement strategies. Several studies report that employees show restraint and consider the QI work to be useless if they feel forced to take part in it [2, 4, 9, 21]. Other studies confirm that positive relationships and aligning QI work with professional logic [22], in conjunction with work structures that empower employees [13], influence how employees engage in QI work. The supervision and learning methods inspired by Illeris’ [24] perspective of transformative learning, which involves changes in the learners’ perspectives as a result of critical reflection and open discourse, might have been an effective contribution to back up the leaders and give them a new understanding in practice. The fact that these FLNMs participated in creating the theoretical perspective for the QIC may have made them more interested and even compelled to undertake QI work.

A hierarchical and linear leadership style seems to no longer apply to the highly complex, interrelated, relationship-driven QI work [4]. Davidson [9] used complex responsive processes (CRPs) (what she called a lens) to understand contemporary healthcare leadership. The CRPs give us a description of organizations as processes of human interaction, which occurs here and now. Traditionally, we are accustomed to considering problem solving in a rational and causal way. CRPs provide a new starting point to solving a problem by focusing on living in the present. It allows us to conceptualize causality in a transformative way, where the future is under perpetual construction, rather than predetermined as in rational causality [9]. According to Bondas [17], nurse managers seemed to have the will, but not always the knowledge, to mold a synthesis of nursing care knowledge in combination with leadership and management knowledge. The transformative perspective of causality in CRPs fit well with knowledge of nursing care in leadership and with Illeris` [24] perspective of transformative learning. The CRPs tell us that choices arise in present moment-to-moment interactions, and the future takes shape through interaction and mutual selection between persons in the living present [9]. In this study, the analysis suggests that the FLNMs solve problems by focusing on living in the present.

Participation in QIC may influence the clinical results of QI work, as shown by the FLNMs’ descriptions of how they used the QIC to master the processes of QI work with the staff. The FLNMs emphasized that continuous improvement depends on how the staff are able to carry this out, and how, as leaders, the FLNMs can prepare the way for the staff. The perceived trust in employee competence has shown to be a prerequisite for creating constructive working relationships between managers and employees, and thus positive attitudes towards and involvement in QI work [21]. The FLNMs in this study established security for the staff to providing good care. Studies confirm that the relationship between leadership, safety, and quality depends on a trusting relationship between nurse leaders and employees [14], and is an important driving force in the achievement of positive patient outcomes [1]. Bondas [17] found that the nurse leaders did not want their staff to regard them as controllers or experts who always knew better. In this study, the leaders considered themselves supervisors who involved and supported the employees. Gadolin and Andersson [21] found that professionalization and work structures may prevent employees from engaging in improvement work, and that reduced involvement in QI work induces resistance and dysfunctional behavior. The FLNMs in this study seemed to better understand that improved patient safety depends on the employees’ competence and capability to adapt their working practices to a better way of working. How the leaders talked about their staff referred to the dignity aspect of the leaders caring for the employees, as an aspect of caritative leadership [18]. A central aspect of caritative leadership is the caritas motive of agape and unconditional human love and mercy when ministering to the patient [19]. The relationship between the leaders and their employees may be motivated by the same interest: ministering to the patient [18]. This caring culture of nursing leadership facilitates the best patient care by trusting the professional staff and their knowledge [16]. The finding in Gadolin and Andersson’s [21] study was perhaps a result of caritative leadership, when they wrote that the way QI efforts are introduced, deployed, and rhetorically driven may affect employee attitudes and engagement. When ministering to patients based on QI efforts you do not need to speak rhetorically. Caritative leadership may, in this way, serve as the ethical conscience of the QI work and help the FLNM focus on the main objective of the unit: nursing care. By recognizing the uniqueness of the employees and their potential for QI work, the leader can enable the core practice at its best [18].

### Limitations

Studying other QIC may have yielded different results. However, the choice of studying this self-developed and voluntary QIC, and the teaching in the general topics of improvement strategies based on transformative learning (as opposed to the classical approach of a QIC), have allowed us to see other perspective of QI work and other organized QIC. This may have influenced the results. The limitations of this study are inherent within the qualitative method [26]. Even though we could report FLNMs’ own perspectives on how participating in the QIC contributed and gave meaning to QI work, the participation in focus groups may limit the credibility and transferability of the findings [28]. Observational studies or individual interviews might have revealed other perspectives [28]. The study‘s confirmability was strengthened by how we, as a team with different perspectives, representing two Nordic countries and various professional backgrounds, worked on the analysis. The study design did not allow us to identify specific QI work practices or elements of managerial responsibility in QI work that influence the clinical results. While this study contributes to a deeper understanding of FLNMs’ experiences, a continuous effort is needed to refine studies of how FLNMs’ participation in the QIC impacts QI work in practice.

### Implications

The results of this study demonstrate that QIC might influence the clinical results of QI work by supporting the FLNMs in their desired goal of improving patient outcomes. This study provides new knowledge about how a QIC can make frames for the FLNMs to handle the complex context for QI work, based on a fellowship facilitating critical thinking to prioritize the patient and securing improvement processes. Supportive elements in the fellowship may promote a way of setting boundaries and understanding leadership requirements in QI work. Collegial collaboration in a QIC seemed help the participants in this study improve their ability to complete QI work, make decisions, and solve problems in their own department. In the same way that Bondas showed QI work supporting development teams required integration of both a caring leadership culture and caring actions through humane and humble leadership, the participants in this study favored the employee’s involvement in the QI work. Furthermore, they drew attention to the importance of patient involvement and underlined that this involvement contributed to the success of the improvement interventions.

## Conclusion

A QIC encompassing knowledge transfer and reflection may create an important fellowship for health care leaders to do quality improvement work in their own organization. It is crucial to invite all leaders from an organization to share the experience and continue their collaboration together with their staff in their own organization. Continuity over time, following elements of the QI work at joint meetings, involvement by users, and self-development of and voluntary involvement in the QIC seem to be important for knowledge development in QI. The supportive elements of the QIC fellowship were crucial to critical thinking and to the FLNMs’ own development and security in mastering the QI work processes. In this way they preferred to prioritize the patients in QI work, despite haste and obstructive situations in an everyday context.

## Supplementary information

**Additional file 1.** Interview guide

## Data Availability

The interview guide is available in Additional file 1. The datasets analyzed during the current study are available from the corresponding author on reasonable request.

## Abbreviations

CRP: Complex responsive processes
FLNM: First-line nurse manager
H: FLNM from the local hospital
HS: FLNM from the municipal homecare services
LTS: FLNM from the municipal long-term care services
QI: Quality improvement
QIC: Quality improvement collaborative

## References

[1] Verschueren M, Kips J, Euwema M (2013). A review on leadership of head nurses and patient safety and quality of care. Adv Health Care Manage.

[2] Elg M, Stenberg J, Kammerlind P, Tullberg S, Olsson J (2011). Swedish healthcare management practices and quality improvement work - development trends. Int J Health Care Quality Assur.

[3] Sirola-Karvinen P, Hyrkäs K (2006). Clinical supervision for nurses in administrative and leadership positions: a systematic literature review of the studies focusing on administrative clinical supervision. J Nurs Manag.

[4] McKimm J, Till A (2015). Clinical leadership effectiveness, change and complexity. Br J Hosp Med.

[5] Øvretveit J, Dolan-Branton L, Marx M, Reid A, Reid J, Agins B (2018). Adapting improvements to context:: when, why and how?. Int J Qual Health Care.

[6] Berwick DM (2011). Preparing nurses for participation in and leadership of continual improvement. J Nurs Educ.

[7] Belrhiti Z, Giralt AN, Marchal B (2018). Complex Leadership in Healthcare: A Scoping Review. (Scoping Review). Int J Health Policy Manag.

[8] Cummings GG, Tate K, Lee S, Wong CA, Paananen T, Micaroni SPM (2018). Leadership styles and outcome patterns for the nursing workforce and work environment: a systematic review. Int J Nurs Stud.

[9] Davidson SJ (2010). Complex responsive processes: a new Lens for leadership in twenty-first-century health care. Nurs Forum.

[10] Øvretveit B, Cleary C, Gustafson MI (2002). Quality collaboratives: lessons from research. Qual Saf Health Care.

[11] Wells S, Tamir O, Gray J, Naidoo D, Bekhit M, Goldmann D (2018). Are quality improvement collaboratives effective? A systematic review. BMJ Qual Saf.

[12] Block LAM, Manning LJ (2007). A systemic approach to developing frontline leaders in healthcare. Leadersh Health Serv.

[13] Hartviksen TA, Aspfors J, Uhrenfeldt L (2019). Healthcare middle managers’ experiences of developing capacity and capability: a systematic review and meta-synthesis. BMC Health Serv Res.

[14] Chisengantambu C, Robinson GM, Evans N (2018). Nurse managers and the sandwich support model. J Nurs Manag.

[15] Rao AD, Evans LK, Mueller CA, Lake ET (2019). Professional networks and support for nursing home directors of nursing. Res Nurs Health.

[16] Bondas T (2018). Self-organizing development teams for innovative nursing care. Nurs Adm Q.

[17] Bondas T (2009). Preparing the air for nursing care: a grounded theory study of first line nurse managers. J Res Nurs.

[18] Bondas T (2003). Caritative leadership: ministering to the patients. Nurs Adm Q.

[19] Bondas T (2010). Nursing leadership from the perspective of clinical group supervision: a paradoxical practice. J Nurs Manag.

[20] Hartviksen TA, Sjolie BM, Aspfors J, Uhrenfeldt L (2018). Healthcare middle managers` experiences developing leadership capacity and capability in a public funded learning network. BMC Health Serv Res.

[21] Gadolin C, Andersson T (2017). Healthcare quality improvement work: a professional employee perspective. Int J Health Care Qual Assur.

[22] Lalani Mirza, Hall Kate, Skrypak Mirek, Laing Chris, Welch John, Toohey Peter, Seaholme Sarah, Weijburg Thomas, Eyre Laura, Marshall Martin (2018). Building motivation to participate in a quality improvement collaborative in NHS hospital trusts in Southeast England: a qualitative participatory evaluation. BMJ Open.

[23] Graneheim UH, Lundman B (2004). Qualitative content analysis in nursing research: concepts, procedures and measures to achieve trustworthiness. Nurse Educ Today.

[24] Illeris K (2015). The development of a comprehensive and coherent theory of learning. Eur J Dent Educ.

[25] Illeris K (2002). Udspil om læring i arbejdslivet (The Fundamentals of Workplace Learning).

[26] Kvale S, Brinkmann S, Anderssen TM, Rygge J (2009). Det kvalitative forskningsintervju (Learning the craft of qualitative research interviewing).

[27] Kamberelis G, Dimitriadis G, Denzin NK, Lincoln YS (2005). Focus groups, contingent articulations of pedagogy, politics, and inquiry. The sage handbook of qualitative research.

[28] Morgan DL (1997). Focus groups as qualitative research.

[29] Norwegian Centre for Research Data (2016). Informal Notification Test Norwegian Data Protection Official for Research.

[30] The Norwegian National Research Etics Committees NNREC (2016). Guidelines for research ethics in the social sciences, humanities, law and theology.

