# Supplementary material for: “Navigation to prioritizing the patient” – first-line nurse managers’ experiences of participating in a quality improvement collaborative
Source: BMC Health Serv Res. 2020 Jan 22;20:55. doi: 10.1186/s12913-020-4918-z (PMC6977232; doi:10.1186/s12913-020-4918-z)
Supplement: Supplementary file 1 — Additional file 1. Interview guide [file 12913_2020_4918_MOESM1_ESM.docx]

**Additional file 1**

**Interview guide**

How would you describe the usefulness of participating in the learning network?

How would you describe your experiences executing leadership before and after participating in the learning network?

Any changes in how you think about leadership?

Any changes in how you perform leadership?

How would you describe your abilities as a healthcare middle manager?

How is this influenced by participating in the network?

How does your participation in the network influence your staff?

How does your participation in the network influence the recipients of your services?

How can you compare these processes with other processes in your life?

Supporting questions: Can you add some examples?

Why does this happen?

How did this happen?

How did you know this?

How could this be changed.
